# Comprehensive characterization of posttranscriptional impairment-related 3′-UTR mutations in 2413 whole genomes of cancer patients

**DOI:** 10.1038/s41525-022-00305-0

**Published:** 2022-06-02

**Authors:** Wenqing Wei, Wenyan Gao, Qinglan Li, Yuhao Liu, Hongyan Chen, Yongping Cui, Zhongsheng Sun, Zhihua Liu

**Affiliations:** 1 State Key Laboratory of Molecular Oncology, National Cancer Center, National Clinical Research Center for Cancer, Cancer Hospital, Chinese Academy of Medical Sciences and Peking Union Medical College, Beijing, 100021 China; 2 Beijing Institutes of Life Science, Chinese Academy of Sciences, Beijing, 100101 China; 3 University of Chinese Academy of Sciences, Beijing, 100049 China; 4 Department of Radiation Oncology, National Cancer Center/National Clinical Research Center for Cancer/Cancer Hospital & Shenzhen Hospital, Chinese Academy of Medical Sciences and Peking Union Medical College, Shenzhen, 518116 China; 5 Department of Oncology, Cancer Institute, Peking University Shenzhen Hospital, Shenzhen Peking University-Hong Kong University of Science and Technology (PKU-HKUST) Medical Center, Shenzhen, 518035 China

**Keywords:** Cancer genomics, Prognostic markers, Predictive markers

## Abstract

The 3′ untranslated region (3′-UTR) is the vital element regulating gene expression, but most studies have focused on variations in RNA-binding proteins (RBPs), miRNAs, alternative polyadenylation (APA) and RNA modifications. To explore the posttranscriptional function of 3′-UTR somatic mutations in tumorigenesis, we collected whole-genome data from 2413 patients across 18 cancer types. Our updated algorithm, PIVar, revealed 25,216 3′-UTR posttranscriptional impairment-related SNVs (3′-UTR piSNVs) spanning 2930 genes; 24 related RBPs were significantly enriched. The somatic 3′-UTR piSNV ratio was markedly increased across all 18 cancer types, which was associated with worse survival for four cancer types. Several cancer-related genes appeared to facilitate tumorigenesis at the protein and posttranscriptional regulation levels, whereas some 3′-UTR piSNV-affected genes functioned mainly via posttranscriptional mechanisms. Moreover, we assessed immune cell and checkpoint characteristics between the high/low 3′-UTR piSNV ratio groups and predicted 80 compounds associated with the 3′-UTR piSNV-affected gene expression signature. In summary, our study revealed the prevalence and clinical relevance of 3′-UTR piSNVs in cancers, and also demonstrates that in addition to affecting miRNAs, 3′-UTR piSNVs perturb RBPs binding, APA and m6A RNA modification, which emphasized the importance of considering 3′-UTR piSNVs in cancer biology.

## Introduction

The 3′ untranslated region (3′-UTR) is a posttranscriptional regulatory region that crucially controls gene regulation, and contains many regulatory elements that regulate a variety of mRNA-fate-related processes, such as mRNA processing, mRNA stabilization, translation initiation, and localization^1,2^. In recent decades, most cancer studies have mainly focused on the variations in miRNA, RNA-binding proteins (RBPs), alternative polyadenylation (APA), and m6A RNA modification on the 3′-UTR of mRNA, but 3′-UTR mutations could also result in mRNA expression changes, and recurrent 3′-UTR mutations in cancer genes have been identified by whole-genome sequencing (WGS) to play vital roles in tumorigenesis^2^. Therefore, it is crucial to explore the effect of 3′-UTR mutations on pathological processes at the posttranscriptional level.

RBPs are involved in all aspects of RNA regulation, including splicing, modification, mRNA stabilization, translation, subcellular localization, and decay^3–5^, while the dysregulation of RBPs could systematically disrupt the stable cellular environment^6^. Aberrant expression of RBPs such as RBM38^7^, HuR^8^, and eIF2B^3,9^ is associated with neurodegenerative disorders and cancer progression. Abnormal expression of cancer-related RBPs has been approved to destroy the posttranscriptional regulation network and contributes to tumorigenesis and cancer progression. For example, EZH2 is an immune-related and prognosis-associated RBP in liver cancer^10^, and LARP1 promotes ovarian cancer tumorigenesis, progression, and chemotherapy resistance^11,12^.

RBPs regulate gene expression and function by binding to sequence-specific binding motifs in RNA^13,14^. In cancer cells, there are many accumulated genetic variants that destroy the protein-RNA interactions binding motifs that prevent RBPs from recognizing RNA substrates^15^. For instance, mutant R521C stabilizes FUS in amyotrophic lateral sclerosis (ALS) patients, facilitating its interaction with RBM45, and decreases the recruitment of HDAC1 to contribute to the pathogenesis of ALS^16^. In particular, RBPs can bind to 3′-UTR cis-elements to regulate gene expression^2^. However, it remains unclear how 3′-UTR single nucleotide variants (SNVs) affect RBP-mediated posttranscriptional regulation in human cancers^6^.

In this study, we comprehensively characterized posttranscriptional regulation by somatic 3′-UTR mutations, which could contribute to tumorigenesis. In total, we characterized over twenty thousand posttranscriptionally impairment-related SNVs (piSNVs) from whole-genome sequencing data (WGS) of 2413 patients across 18 cancer types, via our updated algorithm PIVar. Moreover, we found that somatic 3′-UTR piSNV ratio could be used as a potential prognostic biomarker in various cancers and could assess immune cell and checkpoint characteristics. These 3′-UTR piSNVs could affect genes by mechanisms of posttranscriptional regulation via RBPs, miRNA, APA, and m6A modification, which are involved in many tumor-related pathways to contribute to cancer development^3^.

## Results

### Identification and distribution of 3′-UTR piSNVs across cancers

**Fig. 1:**
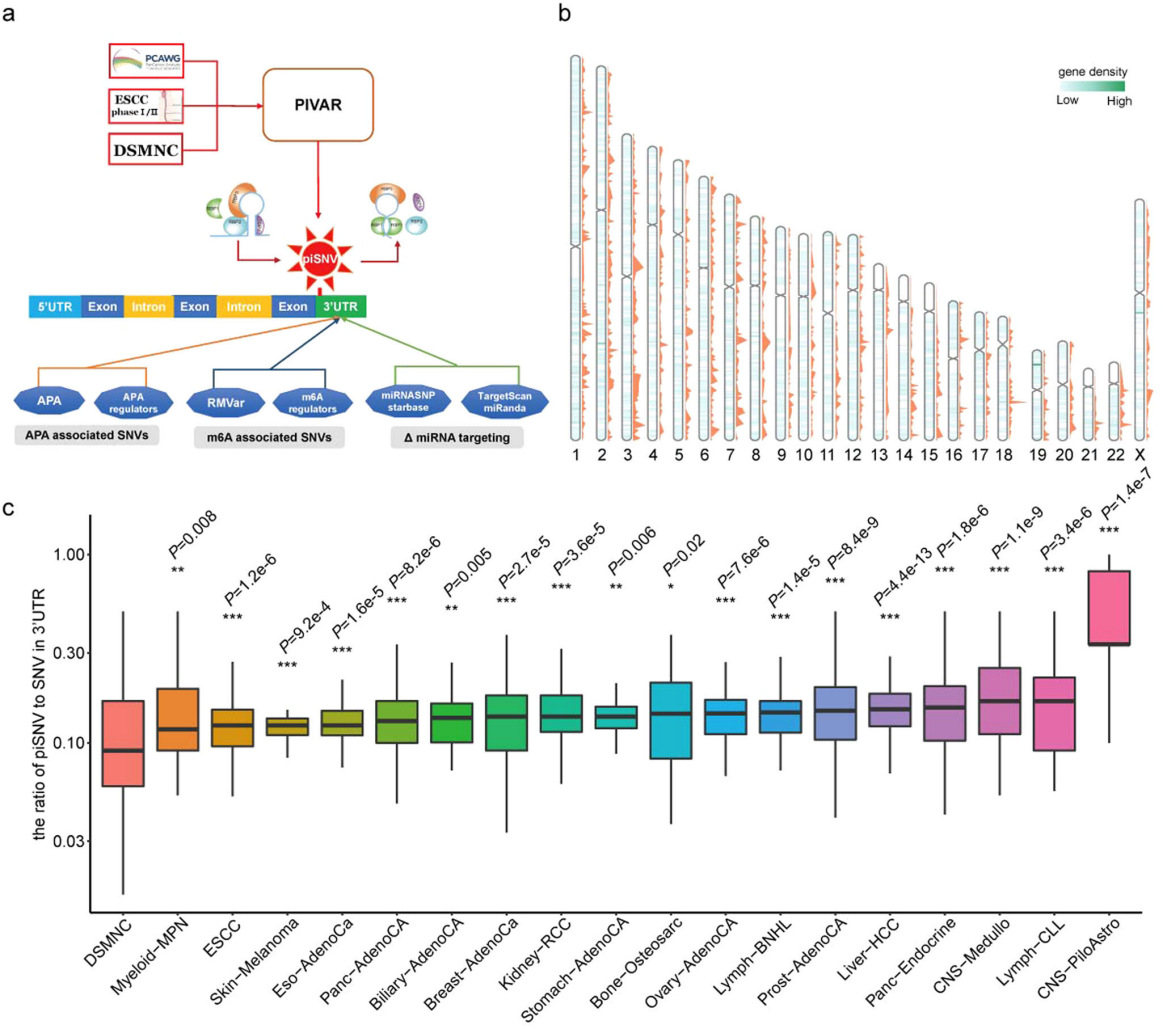
3′-UTR post-transcriptional impaired SNVs (3′-UTR piSNVs) identified in PCAWG project and ESCC cohorts. **a** General workflow for identifying 3′-UTR post-transcriptional impaired SNVs. **b** Genome-wide gene density distribution of identified piSNVs (blue lines in chromosome body) and 3′-UTR piSNVs (orange peaks) in each chromosome. **c** Compared to controls in the DSMNC database, the proportion of somatic 3′-UTR piSNVs was significantly elevated in the PCAWG project and ESCC cohorts (*P < 0.05, **P < 0.01, ***P < 0.001). We calculated the ratio of piSNV to SNV in 3′-UTR for each sample, and used wilcoxon rank-sum test to evaluate the distribution differences of 3′-UTR piSNV ratio between cancer samples in each cancer type and control samples. Boxplots elements represent: center line = median, upper and lower hinges = 25 and 75% percentiles, upper and lower whisker = mean ± 1.5*IQR.

RBPs play vital roles in regulating the mRNA life cycle by binding 3′-UTRs^2,3^. To evaluate the potential impact of 3′-UTR mutations on posttranscriptional regulation in tumorigenesis, we downloaded somatic mutations derived from WGS data of 2413 patients across 18 cancer types in the PCAWG project^17^, including biliary adenocarcinoma (Biliary-AdenoCA), osteosarcoma (Bone-Osteosarc), breast adenocarcinoma (Breast-AdenoCa), medulloblastoma (CNS-Medullo), pilocytic astrocytoma (CNS-PiloAstro), esophagus adenocarcinoma (Eso-AdenoCa), kidney renal cell carcinoma (Kidney-RCC), liver hepatocellular carcinoma (Liver-HCC), lymphoid mature B-cell lymphoma (Lymph-BNHL), lymphoid chronic lymphocytic leukemia (Lymph-CLL), myeloid myeloproliferative neoplasm (Myeloid-MPN), ovarian adenocarcinoma (Ovary-AdenoCA), pancreatic adenocarcinoma (Panc-AdenoCA), pancreatic neuroendocrine tumor (Panc-Endocrine), prostate adenocarcinoma (Prost-AdenoCA), skin melanoma (Skin-Melanoma) and stomach adenocarcinoma (Stomach-AdenoCA), as well as our in-house esophageal squamous cell carcinoma (ESCC) WGS samples (663 patients)^18^, then we employed an updated PIVAR algorithm^6^ that could identify the variants that disrupt protein-RNA interaction via the alteration of RNA secondary structure and the regulation of gene expression; we identified 25,216 3′-UTR piSNVs among 50,435 piSNVs from 1750 samples of the PCAWG project^17^ and 663 samples from our in-house ESCC WGS data^18^ across 18 cancer types (Fig. 1a).

By comparing the genome-wide gene density distribution of identified piSNVs and 3′-UTR piSNVs (orange peaks) in each chromosome, we observed that piSNVs and 3′-UTR piSNVs were clustered in some specific chromosome regions, while there were certain desert regions for piSNVs and 3′-UTR piSNVs (Fig. 1b). Among the genomic regions, 3′-UTR piSNVs were generally more significantly enriched in the 3′-UTR and exon regions in all 18 cancer types (Supplementary Fig. 1a, b), which suggested the SNVs in the 3′-UTR and exon regions have more regulatory functions. We further calculated the ratio of 3′-UTR piSNVs to total 3′-UTR SNVs in each sample across cancer types and found a significantly higher ratio of 3′-UTR piSNVs in pan-cancer samples compared with that of the control samples from the Database of Somatic Mutations in Normal Cells (DSMNC)^19^ (Fig. 1c). The significant distinction between cancer samples and normal controls revealed the prevalence of posttranscriptionally impairment-related 3′-UTR mutations in cancer genomes, implying their contribution to cancer development.

To further explore the association between 3′-UTR piSNVs and cancers, we downloaded GWAS SNPs data from GWAS Catalog database^20^ and CCGD-ESCC database^21^, and we found that there were two 3′-UTR piSNVs, rs63629260 and rs3767, overlapping with GWAS SNPs from GWAS Catalog database; rs63629260 is the 3′-UTR mutation in *SPTBN1* and has association with bone mineral density, osteoporosis and fracture^22^; and rs3767 is the 3′-UTR mutation in *ZNF664* and has relation with morphogenesis, organogenesis, adrenal cell renewal, and cancer^23^. Moreover, there were 121 3′-UTR piSNVs existing in ESCC GWAS data of CCGD-ESCC database (Supplementary Table 1), which suggested that posttranscriptional regulation by somatic 3′-UTR mutations could contribute to tumorigenesis.

### Clinical relevance and immune effect of an elevated 3′-UTR piSNV ratio

**Fig. 2:**
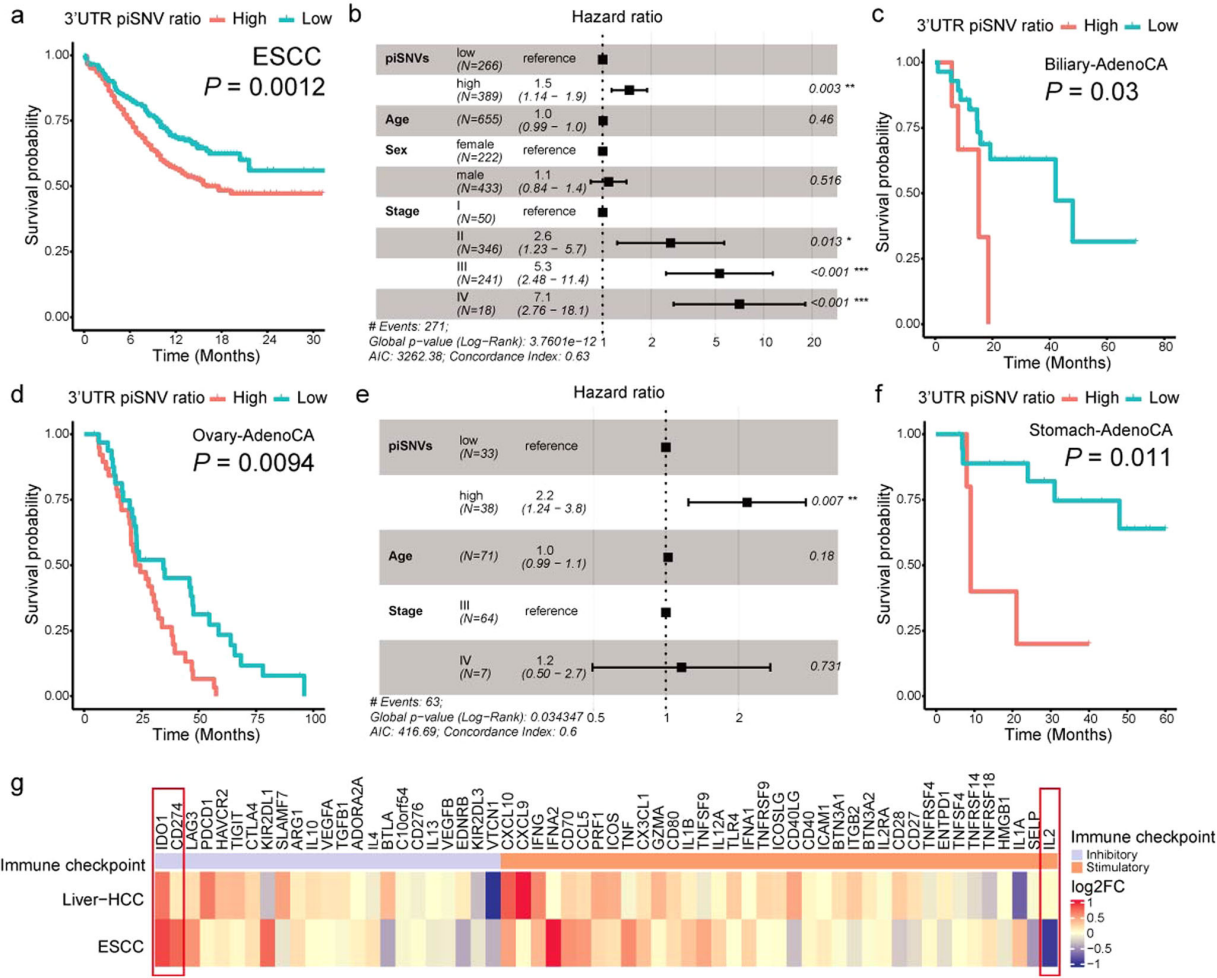
Overall survival and expression of immune checkpoint genes related to the 3′-UTR piSNV ratio across cancer types. Kaplan–Meier curves displayed overall survival of patients with high- (red) and low- (red) 3′-UTR piSNV ratio in ESCC (**a**), Biliary-AdenoCA (**c**), Ovary-AdenoCA (**d**), and Stomach-AdenoCA (**f**). Multivariate Cox regression analysis of the 3′-UTR piSNV ratio in ESCC (**b**) and Ovary-AdenoCA (**e**), which included the factors of age, gender, and TNM stage of patients. **g** Tumor expression difference of immune checkpoint genes between the high- and low-3′-UTR piSNV ratio groups in ESCC and Liver-HCC, fold changes were the ratios of the tumor sample in high-3′-UTR piSNV ratio group to low-3′-UTR piSNV ratio group.

To assess the correlation between the 3′-UTR piSNV ratio and clinical phenomena, we classified tumor samples according to the 3′-UTR piSNV ratio and performed survival analysis. We found that the overall survival (OS) of patients with four cancer types, including ESCC (Fig. 2a, b), Biliary-AdenoCA (Fig. 2c), Ovary-AdenoCA (Fig. 2d, e), and Stomach-AdenoCA (Fig. 2f), had a significant correlation with the 3′-UTR piSNV ratio. Moreover, the patients with a high 3′-UTR piSNV ratio in each cancer type had a worse survival situation than the patients with a low 3′-UTR piSNV ratio. These results further showed that an increased 3′-UTR piSNV ratio is related to poor clinical outcomes and implied that the 3′-UTR piSNV ratio could function as a potential prognostic index in several types of cancers.

We speculated whether immune microenvironment changes could contribute to survival outcomes. Then, we used the CIBERSORT^24^ algorithm to compute the relative abundance of 22 types of immune cells (Supplementary Fig. 2a–d). Since the samples for WGS and RNA-seq were unpaired, we finally selected ESCC and Liver-HCC datasets, which had enough samples in both the high and low 3′-UTR piSNV groups, to calculate immune cell abundance. Overall, there was an obvious difference in immune cell enrichment between the high- and low-3′-UTR piSNV ratio groups. Notably, the level of resting NK cells was significantly higher in the high-3′-UTR piSNV ratio group in ESCC (Supplementary Fig. 2a, b); however, the low-3′-UTR piSNV ratio group of the Liver-HCC cohort showed significantly higher levels of resting NK cells (Supplementary Fig. 2c, d). Moreover, type 2 macrophages [M2], as immunoinhibitory cells, were more enriched in the high-3′-UTR piSNV ratio group (Supplementary Fig. 2c, d).

Immune checkpoints are essential for the immune response and can result in cancer cell immune escape, and immune checkpoint inhibitors, in the form of blocking antibodies, are applied to facilitate an immune response in cancers^25–28^. We further explored the expression difference in immune checkpoint genes between the high- and low-3′-UTR piSNV ratio groups in tumor samples (Fig. 2g), and we found that *IDO1* and *CD274* were inhibitory immune checkpoints with significantly higher expression in high-3′-UTR piSNV ratio groups of ESCC and Liver-HCC (Supplementary Fig. 2e, f). *IDO1* is a target for cancer immunotherapy and encodes a heme enzyme that acts on multiple tryptophan substrates and plays a crucial role in pathophysiological processes such as immunoregulation, antitumor defense, and antioxidant activity^29,30^. CD274 (PD-L1) is a well-known ligand that binds with the receptor PD1 in T cells, which can block T-cell activation in cancer^31^. In addition to the immune inhibitors, we also observed that the immune stimulator *IL2* was significantly decreased in the high-3′-UTR piSNV ratio group of ESCC (Supplementary Fig. 2e). IL2 is a primary cytokine for T and NK cell proliferation and activation and affects immune homeostasis by regulating regulatory T (Treg) cells, which play a crucial role in immune cancer therapy^32,33^. These results suggested that 3′-UTR piSNVs are involved in immune microenvironment heterogeneity and induce the dysregulation of several core immune regulators, which contributes to worse clinical outcome.

### Functional effect of 3′-UTR piSNVs on RBP binding

**Fig. 3:**
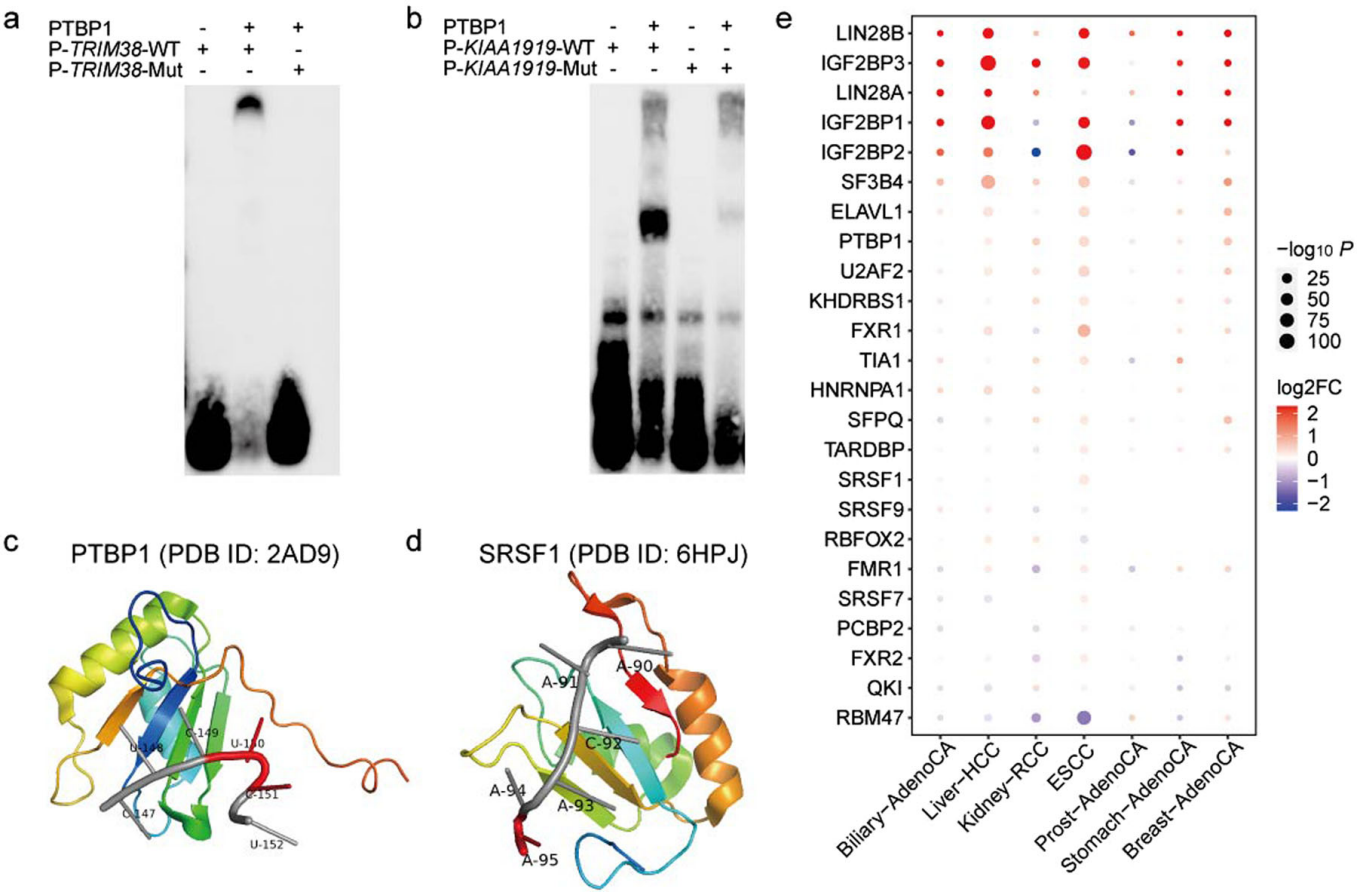
The functional effects of 3′-UTR piSNVs on RBP binding. Electrophoretic mobility shift assays (EMSA) results show the binding impact of 3’-UTR piSNVs of *TRIM38* (**a**) and *KIAA1919* (**b**) on the recognition of PTBP1 to their RNA targets. Crystal structure of the PTBP1 (**c**; PDB ID: 2AD9) or SRSF1 (**d**; PDB ID: 6HPJ) in complex with specific RNA motifs, red region on RNA oligo presented the mutated sites. **e** Expression of 24 enriched RBP motifs of 3′-UTR piSNVs in different cancer types.

Genetic mutations on RNA substrates can destroy the protein–RNA interactions binding motifs to prevent RBPs from recognizing RNA substrates in cancer cells^15^. To access the experimental effect of these 3′-UTR piSNVs on RBP binding, we performed electrophoresis mobility shift assays (EMSA) on four randomly selected 3′-UTR piSNVs who predicted to alter the binding of PTBP1 (Fig. 3a, b; Supplementary Fig. 3a, b). As shown in Fig. 3a, there was the strong binding between PTBP1 and unmutated RNA probe of *TRIM38* that is associated with Fanconi renotubular syndrome^34^, while it had visible differences in the probe with the 3′-UTR piSNV of *TRIM38* in their binding to PTBP1. There were similar observations existing in 3′-UTR piSNV of *KIAA1919*, *CNTLN,* and *MOB3B* on binding of PTBP1 (Fig. 3b; Supplementary Fig. 3a, b).

We further examined the effect of 3′-UTR piSNVs on RBP binding with the PRIdictor^35^ webtool, which can predict the binding changes between mutual binding sites in RNA and protein, and we found that 3′-UTR piSNVs could destroy the PTBP1-RNA (Fig. 3c, PDB ID: 2AD9) and SRSF1-RNA (Fig. 3d, PDB ID: 6HPJ) binding complex. In detail, PTBP1 can bind to 5′-CUCUCU-3′ RNA oligonucleotides^36^. By screening the identified 3′-UTR piSNVs, we found that 21 genes had a mutation from C to T at the fifth position of the above motif mainly in Skin-Melanoma and ESCC (Fig. 3a, Supplementary Fig. 3c, and Supplementary Table 2). Another RBP affected by a 3′-UTR piSNV is SRSF1, which plays an important role in the regulation of alternative splicing events. Since SRSF1 can bind to the 5′-AACAAA-3′ RNA oligonucleotide (PDB ID: 6HPJ)^37^, we speculated that 3’-UTR piSNVs in the AACAAA sequence motif residing in *CHRM3* mRNA could disrupt the binding between SRSF1 and their corresponding RNAs (Fig. 3b, Supplementary Fig. 3d, and Supplementary Table 3).

To determine the effect of these 3′-UTR piSNVs on RBP binding, we integrated RBP motif and CLIP-seq-derived RBP binding data^38,39^ for all identified 3’-UTR piSNVs, which revealed 24 significantly enriched RBPs. Abnormal expression of RBPs leads to tumorigenesis^3^, so we detected the differentially expressed RBPs between tumor and corresponding normal tissues in different cancer types (Fig. 3e). The results proved that dysregulation of these RBPs occurs broadly in seven cancer types. Consistent with previous studies^40,41^, the RBPs PTBP1 and SRSF1 were differentially upregulated in 6 and 3 cancer types, respectively. Moreover, LIN28A/LIN28B and IGF2BP1/2/3 were the most differentially upregulated RBPs in over 5 cancer types. Moreover, IGF2BP1/2 were significantly downregulated in Kidney-RCC and Prost-AdenoCA. LIN28A/LIN28B are LIN-28 family members that are associated with the developmental timing and self-renewal of embryonic stem cells, and their aberrant expression is related to cancer progression^42^. IGF2BP1/2/3 encode members of the insulin-like growth factor 2 mRNA-binding protein family, such as insulin-like growth factor 2 (IGF2), and bind to the mRNAs of several vital genes, and regulate translation. The dysregulation of IGF2BP1/2/3 was found to be associated with skin squamous cell carcinoma^43^ and pancreatic cancer^44^. Thus, these results suggested that the identified 3′-UTR piSNVs affect the binding of some cancer-associated RBPs in cancer development.

### Effect of 3′-UTR piSNVs on miRNA binding

At the posttranscriptional level, miRNAs play an irreplaceable role in regulating mRNA expression and controlling many biological processes^12,45^. Moreover, 3′-UTR piSNVs may also influence posttranscriptional regulation by affecting miRNA binding. By using TargetScan^46^, miRNASNP^47^, starBase^48^, and miRDB^45^ software to predict the binding miRNA of 3′-UTR piSNVs, we identified a total of 1737 miRNAs that could bind to 1035 3′-UTR piSNV-affected genes. Most recurrent miRNAs are miR-3163 (21), miR-340-5p (19) and miR-186-5p (18), regulating over 18 targeted 3′-UTR piSNV-affected genes.

**Fig. 4:**
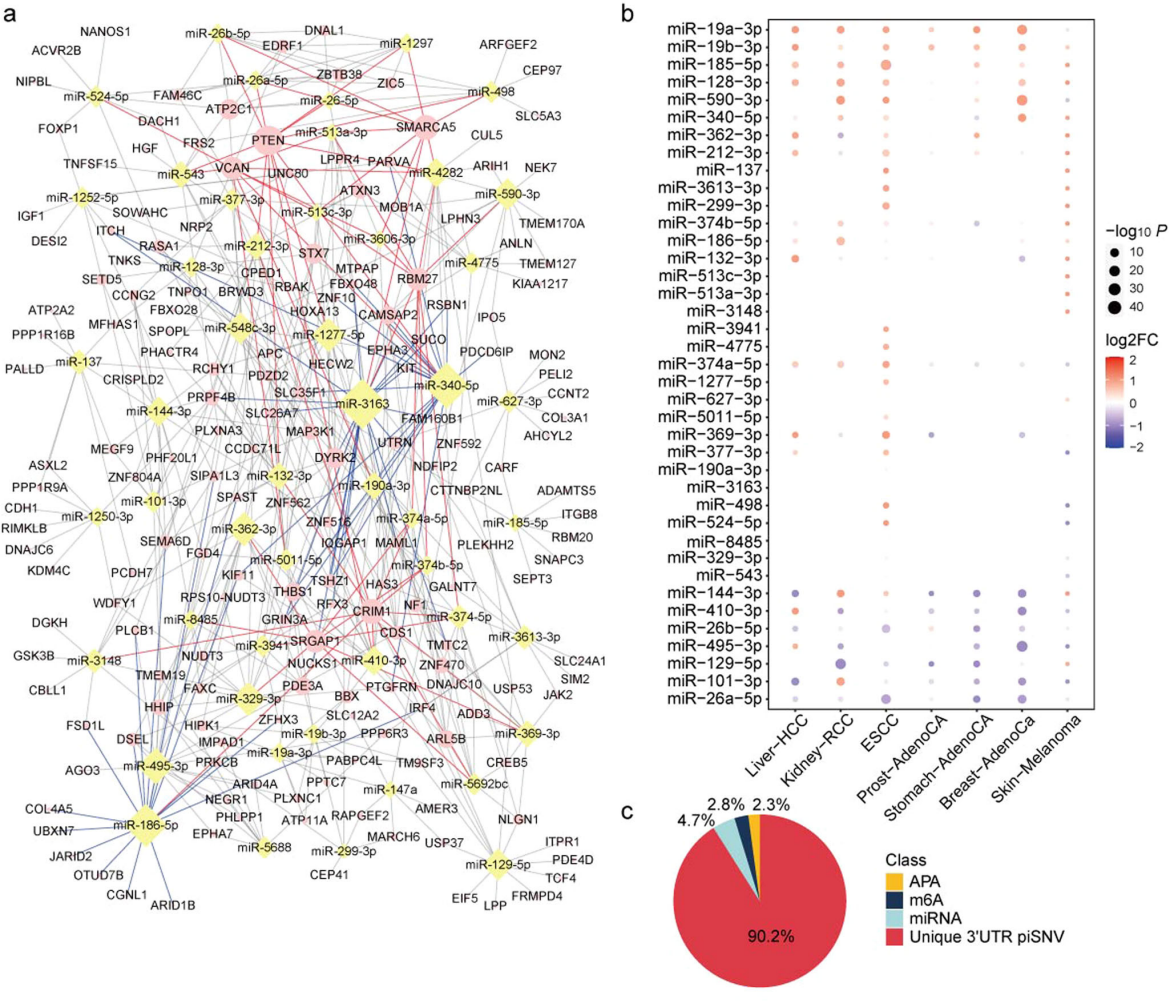
The effect of post-transcriptional regulation on miRNA/m6A/APA. **a** Interaction network between top 50 recurrent binding miRNAs and 3’-UTR piSNV genes, blue lines are the edges of top 3 recurrent binding miRNAs (miR-3163, miR-340-5p and miR-186-5p), and red lines are the edges of the major targeted 3′-UTR piSNV genes (*PTEN*, *CRIM1*, *SMARCA5*, *VCAN*, *SRGAP1,* and *RBM27*). **b** Expression of top 50 recurrent binding miRNAs in different cancer types. **c** The quantitatively distribution of 3′-UTR piSNV in APA, m6A, and miRNA binding sites.

Network illumination of miRNA and the top 50 differential expressed miRNAs showed that several 3′-UTR piSNV-affected genes are regulated by multiple miRNAs (Fig. 4a and Supplementary Table 4). These genes included *PTEN*, *CRIM1*, *SMARCA5*, *VCAN*, *SRGAP1*, and *RBM27*, which may be regulated by over 10 kinds of miRNAs. For example, *PTEN* is a tumor suppressor gene that is commonly lost in human cancer, and is associated with prostate cancer, glioblastoma, endometrial cancer, lung cancer and breast cancer to varying degrees^49,50^. We also observed that the majority of recurrent miRNAs have abnormal expression in cancers. Briefly, 5 miRNAs were significantly differentially upregulated in six kinds of cancers, while 6 miRNAs were downregulated in over five kinds of cancers (Fig. 4b). Our results suggested that several miRNAs show the same dysregulated expression patterns in cancers.

### Predicted APA and RNA modification change of 3′-UTR piSNV sites

The 3′-UTR is a unique area of each gene that bind many functional elements, affect transcript isoform generation, and contain RNA modifications, such as APA and m6A RNA modification. To further explore the effect of 3′-UTR piSNVs on APA changes and m6A modification of transcripts, we annotated 3′-UTR piSNVs with RNA modification and APA changes by RMVar^51^ and APADB^52^. The results showed that 2.8% (628) and 2.3% (515) of 3′-UTR piSNVs had m6A RNA modification and APA changes, respectively (Fig. 4c), occurring mainly in the *SLC16A10, LPGAT1*, *PRKCB*, and *USP38* genes.

We further considered the effect of m6A regulators on the m6A RNA modification of transcripts, and we found that IGF2BP1/2/3, as m6A readers, had significant differential expression in seven cancer types. IGF2BP1/2/3 are IGF2 mRNA-binding proteins that enhance mRNA transcript stability by recognizing m6A featured sequences^53^, and are associated with many cancers, including skin squamous cell carcinoma, testicular cancer, and enchondroma. IGF2BP2 functions as a well-known tumor suppressor gene in lymphoma^54^. We also paid attention to APA regulator expression changes due to 3′-UTR piSNVs and observed widespread dysregulated expression of APA regulators. Most of these APA regulators were specifically upregulated in seven cancer types, while *PPP1CB*, *PCF11*, and *PABPC1* were significantly downregulated in ESCC (Supplementary Fig. 4). These results suggested that variants of 3′-UTR piSNVs and the dysregulation of m6A RNA modification and APA regulators could cause posttranscriptional impairment in mRNA transcripts to contribute to cancer pathogenesis.

### The cooccurrence of 3′-UTR piSNV-affected genes across 18 cancer types

**Fig. 5:**
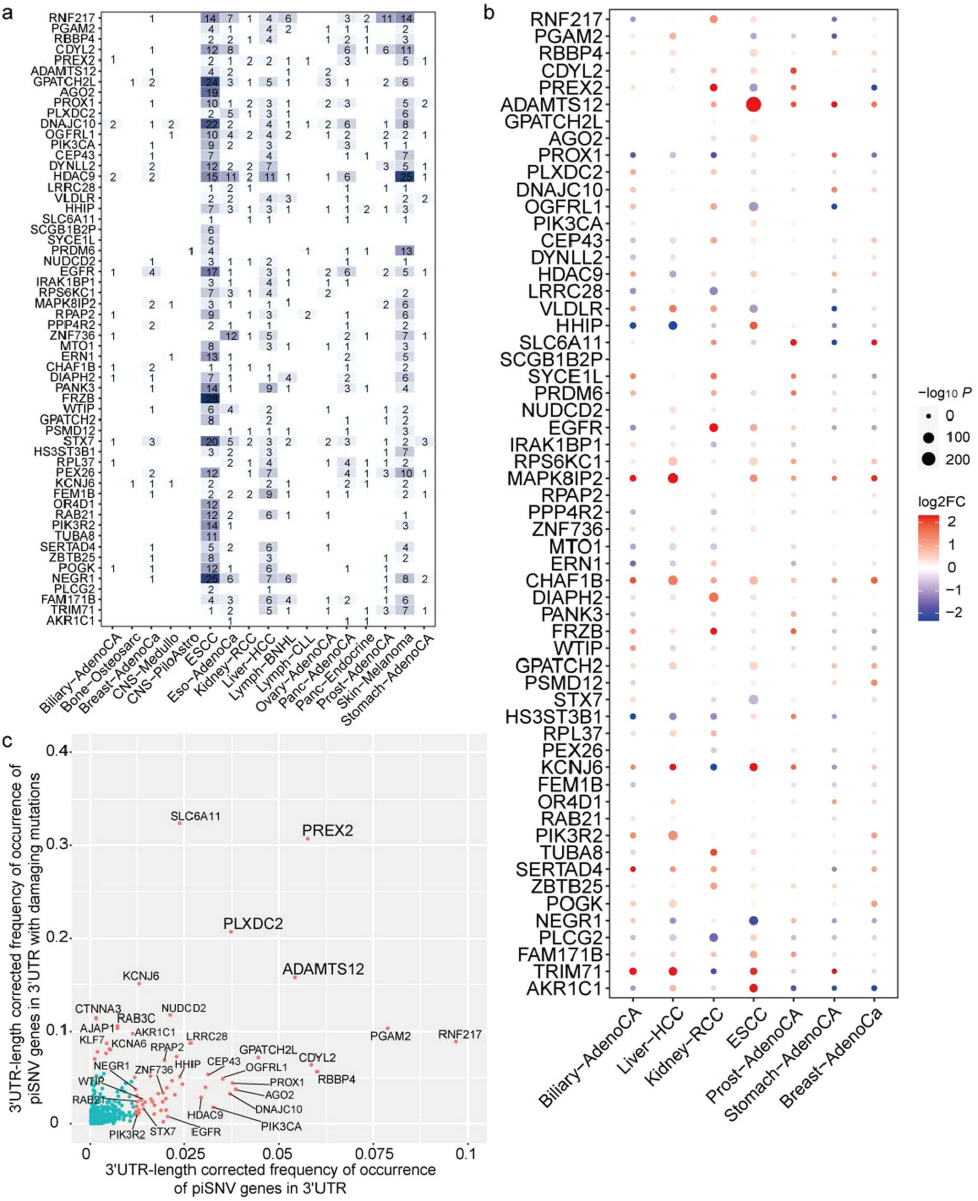
Top 3′-UTR piSNV-affected genes in PCAWG and ESCC cohorts. The cooccurrence (**a**) and expression (**b**) of the top 2% (58) 3′-UTR-length corrected frequently occurred 3′-UTR piSNV-affected genes in cancers. **c** The scatter plot displayed the 3′-UTR-length corrected occurrence frequency of genes at protein and post-transcriptional regulation levels.

To investigate the influence of genes affected by 3′-UTR piSNVs, we reviewed the somatic 3′-UTR piSNVs in each cancer type and observed many tumor-specific genes in 2930 nonredundant 3′-UTR piSNV-affected genes. The top 3′-UTR piSNV-affected genes were *RNF217*, *HDAC9*, *GPATCH2L*, *DNAJC10*, *EGFR*, *PROX1*, *OGFRL1*, and *HHIP*, and variations in these genes arisen in at least 10 cancer types (Fig. 5a). The 3′-UTR piSNVs in gene *RNF217* occurred in 63 patients across 10 cancer types. The RNF217 protein is a member of RING1-IBR-RING24 (RBR) ubiquitin protein ligase family, which contains a transmembrane domain and regulates apoptosis signaling by interacting with HAX1 to promote leukemia development^55^. In total, 70 and 33 patients (among 2413 total patients) had 3′-UTR piSNVs of HDAC9 and EGFR, respectively. The HDAC9 protein is a member of the histone deacetylase family that can repress transcriptional regulation by catalyzing acetyl group removal from lysine residues and is associated with cutaneous T cell lymphoma and maxillary cancer^56,57^. Epidermal growth factor receptor (EGFR) is a receptor tyrosine kinase that contains an extracellular ligand-binding domain and binds to epidermal growth factor, thus inducing receptor dimerization and tyrosine autophosphorylation leading to motility, growth, cell proliferation, and the development of many types of cancer^58,59^. EGFR is an oncogene that is widely amplified and mutated in several cancer types, including non-small cell lung cancer, glioblastoma, and basal-like breast cancers^60^. The T790M EGFR variant has been shown to confer resistance to several drugs, such as gefitinib and erlotinib, and acts as a resistance marker^61,62^. Our data indicated that 3′-UTR variations in HDAC9 and EGFR may promote cancer development through posttranscriptional regulation.

The transcriptome data from the PCAWG project and our in-house ESCC cohort were analyzed to estimate the differential expression of these 3′-UTR piSNV-affected genes between tumor and corresponding normal tissues in seven cancer types. We found that the majority of the top 5% (58) most frequent 3′-UTR piSNV-affected genes were differentially expressed in at least one cancer type (Fig. 5b). For example, *MAPK8IP2*, *ADAMTS12,* and *CHAF1B* were upregulated in over five cancer types, while *HHIP*, *PROX1,* and *PLCG2* were downregulated in over five cancer types.

Then, we investigated the 3′-UTR-length corrected frequency of 3’-UTR piSNV occurrence in 859 genes at the protein function and/or posttranscriptional regulation levels. We found that *PREX2, ADAMTS12*, and *PLXDC2* were the genes most frequently affected by 3′-UTR piSNVs genes in terms of both posttranscriptional changes and functional damage (Fig. 5c). *PREX2* is a member of the phosphatidylinositol 3,4,5-trisphosphate (PIP3)-dependent Rac exchanger (PREX) family. The domain of *PREX2* interacts with the phosphatase and tensin homolog (PTEN) gene product to inhibit PTEN phosphatase activity, thus activating the phosphoinositide-3 kinase (PI3K) signaling pathway, which plays a role in insulin signaling pathways. *PREX2* mutation or overexpression has been observed in some cancers^63,64^. And we found there were both missense mutations and 3′-UTR piSNVs of *PREX2* from the same sample in 5 samples of Skin-Melanoma and 1 sample of Panc-AdenoCA. Interestingly, there was one Skin-Melanoma sample having four different missense mutations and 2 different 3′-UTR piSNVs of *PREX2*, who had higher levels of malignancy (IV, AJCC 7th Edition 2010), while other samples normally had simply one missense mutation or 3′-UTR piSNV. Thus, we inferred that *PREX2, ADAMTS12*, and *PLXDC2* mutations lead to cancer development at both the protein function and posttranscriptional regulation levels. And some identified 3′-UTR piSNV-affected genes, such as *SLC6A11*, *NUDCD2*, *CTNNA3*, *RAB3C,* and *AJAP1*, are well-studied cancer/disease related genes with a high deleterious mutation rate. Intriguingly, we found that some 3′-UTR piSNV-affected genes, such as *RNF217*, *PGAM2*, *RBBP4*, *PIK3CA*, *CDYL2*, *EGFR,* and *PIK3R2*, involved in carcinogenesis mainly via posttranscriptional regulation. The clarification of the features of these 3′-UTR piSNV-affected genes greatly broadened our understanding of cancer biology.

### Functional enrichment and pathological network/pathway analysis of 3′-UTR piSNV-affected genes

**Fig. 6:**
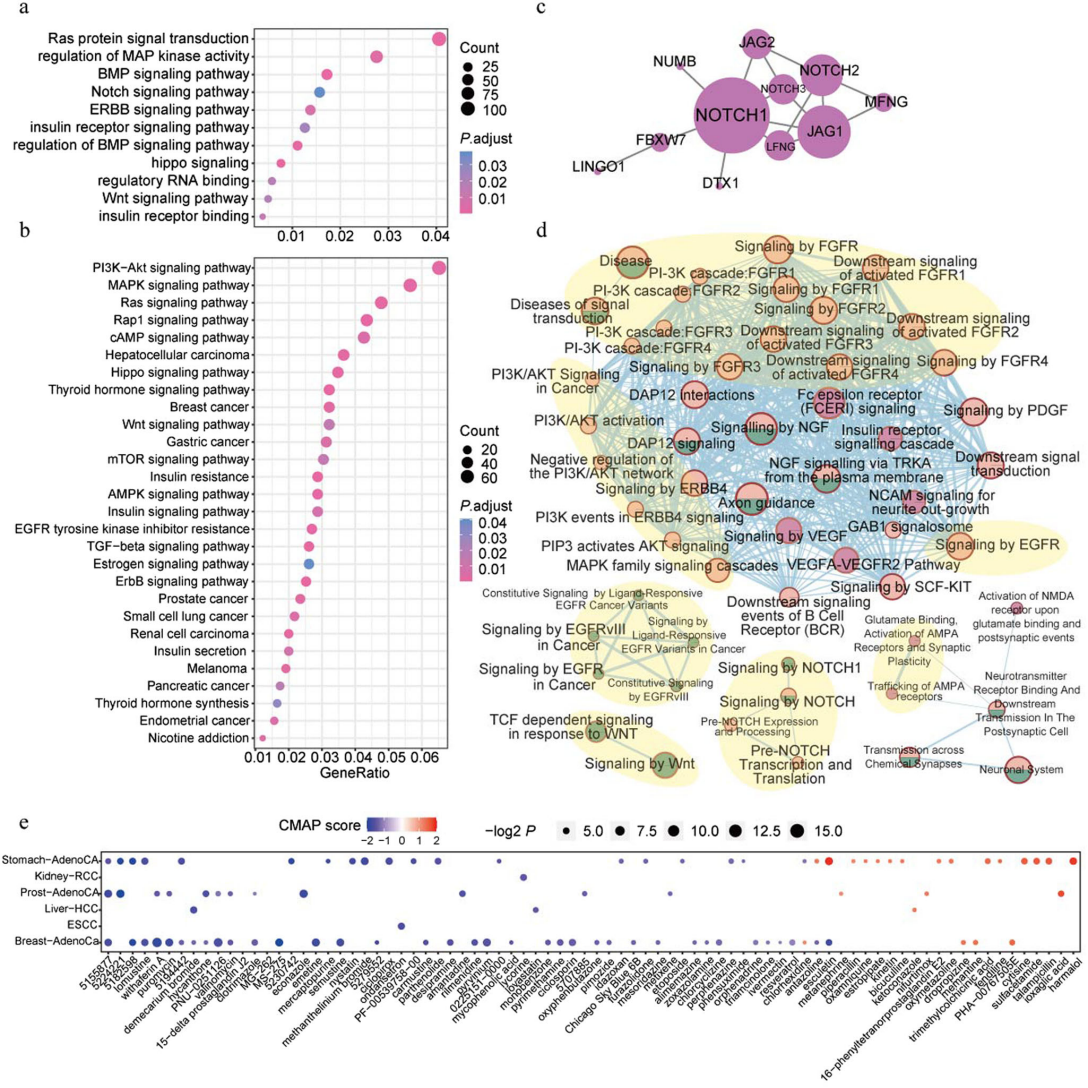
Systematic functional effect of 3′-UTR piSNV genes. GO (**a**) and KEGG (**b**) functional enrichment of 2930 3′-UTR piSNV genes; the significant pathological networks (**c**) and pathways (**d**, pink: 3′-UTR piSNVs; green: damaging mutations; orchid: 3’-UTR piSNVs and damaging mutations; pink and green: 3′-UTR piSNVs or damaging mutations) of 3′-UTR piSNV genes were performed by Hotnet2 and activePathway workflow, respectively. The major signaling pathways (PI3K/AKT/FGFR/MAPK signaling, EGFR-related signaling, AMPA-related signaling, Notch signaling, and Wnt signaling pathways) were pointed out by light yellow circular backgrounds (**d**). **e** Potential clinical drugs identified by CMAP.

To explore the systematic function of 3′-UTR piSNV-affected genes, we used the ‘clusterProfiler’ R package^65^ to perform functional enrichment analysis and found that 3′-UTR piSNV-affected genes were enriched in some canonical cancer pathways and metabolism-related pathways, such as the Notch/Wnt/PI3K-Akt/MAPK/Ras/Hippo/EGFR/ERBB signaling pathways and insulin/thyroid hormone/estrogen pathways (Fig. 6a, b). Then, we applied the Hotnet2^66^ and activePathway^67^ workflows to identify pathological networks and pathways in the HINT + HI2012^68,69^, iRefIndex^70^, and Multinet^71^ protein-protein interaction networks, and we identified 1 significantly altered network and 5 pathological pathways based on the occurrence frequency of each 3′-UTR piSNV gene. The network consisted of *NOTCH1*, *NOTCH2*, *NOTCH3*, *JAG1*, *JAG2*, *LFNG*, *MFNG*, *NUMB*, *FBXW7,* and *LINGO1* (Fig. 6c). These genes are involved in the NOTCH signaling pathway, which controls essential cellular processes, such as proliferation, differentiation and branching morphogenesis^72,73^. Moreover, five pathological pathways, including the PI3K/AKT/FGFR/MAPK signaling, EGFR-related signaling, AMPA-related signaling, Notch signaling and Wnt signaling pathways, had a high correlation with tumor development (Fig. 6d). The above pathological networks and pathways showed that 3′-UTR piSNV-affected genes were involved in vital tumorigenesis processes at the posttranscriptional level.

### Therapeutic implications of 3′-UTR piSNVs and 3′-UTR piSNV-affected genes

To investigate the therapeutic implications of these identified 3′-UTR piSNVs, we employed the Connectivity Map (CMAP) workflow^74^ to assess gene expression profiles for 1309 compounds to identify clinical drugs based on the expression signature of these 3′-UTR piSNV-affected genes. Finally, we identified a total of 80 compounds, and the majority of them were found to have an effect on a specific cancer type (Fig. 6e). 5155877 (HRF-3) was identified as a highly ranked small molecule that affects gene expression signatures associated with 3 cancer types (Fig. 6e). Overall, these identified drugs and compounds might show potential benefits in treating patients with specific types of cancer.

## Discussion

The 3′-UTR can drive or enhance cancer pathogenesis at the posttranscriptional gene regulation level by disrupting regulatory element binding and dysregulating oncogenic gene expression^1^. Previous cancer genomics studies have mostly focused on genetic aberrations of protein-coding regions. WGS of large tumor samples in the PCAWG project and our in-house ESCC cohorts provided an opportunity to explore the comprehensive effect of somatic 3′-UTR mutations on posttranscriptional regulation in cancer. In previous work, we had proved via experimental and computational analyses that somatic mutations identified by PIVar algorithm could disrupt the binding of the RBP-RNA complex^6^.

By employing updated PIVar, we identified 25,216 3′-UTR piSNVs spanning 2930 genes across 18 cancer types and observed the striking phenomenon that most SNVs were 3′-UTR piSNVs, which implied that RBPs prefer to bind in 3′-UTR rather than other regions to modulate posttranscriptional regulation. We compared cancer and control samples from the DSMNC database, and observed an elevated ratio of somatic 3′-UTR piSNVs in pan-cancer, in addition, a high ratio of 3′-UTR piSNVs indicated poor patient survival for four cancer types (ESCC, Ovary-AdenoCA, Biliary-AdenoCA and Stomach-AdenoCA), and there was still an association between the 3’-UTR piSNV ratio and clinical survival in ESCC and Ovary-AdenoCA even when adjusting for other variables by multivariate Cox regression. These results suggested that the 3′-UTR piSNV ratio could be a potential prognostic biomarker for these four kinds of cancers. Notably, some identified 3′-UTR piSNV-affected genes, such as *PREX2, ADAMTS12*, and *PLXDC2*, were regarded as cancer-related genes with a high deleterious mutation rate. For example, *PREX2* is an oncogene that interacts with *PTEN* gene product to inhibit PTEN phosphatase activity, thus activating the PI3K signaling pathway, which plays a role in insulin signaling pathways, and its mutations or overexpression have been observed in some cancers. *PREX2* had the second highest 3′-UTR-length corrected frequency of 3’-UTR piSNV occurrence, and 9 of 2413 cancer patients were also identified to carry protein damaging mutations of *PREX2*; Thus, the 3′-UTR piSNV-affected genes were able to promote carcinogenesis at both the protein function and posttranscriptional regulation levels. Thrillingly, we found that some 3′-UTR piSNV-affected genes, such as *URB1*, *PIK3CA*, *ROBO1*, and *FAT4*, might exert their function during carcinogenesis mainly via posttranscriptional mechanisms. The identification of these 3′-UTR piSNV-affected genes that were not previously well characterized at the posttranscriptional level, which greatly broadened our understanding of cancer biology. In this study, we only focused on the impaired structure and posttranscriptional regulation of 3′-UTR piSNV-affected genes, while some genes may function as trans-acting factors or epigenetic regulators and perform their function at the transcriptional level; thus, future studies need to comprehensively explore the functions of these identified 3′-UTR piSNV-affected genes.

It is unclear how posttranscriptional regulation affects the tumor microenvironment, which determines the invasiveness of cancers^75^. We found that in the worse clinical outcome samples that had a high-3′-UTR piSNV ratio, there was distinct heterogeneity of immune cells in different cancer types, and type 2 macrophages [M2], as immunoinhibitory cells, were more enriched in the high-3′-UTR piSNV ratio groups (Supplementary Fig. 2c, d). Moreover, the inhibitory immune checkpoint *IDO1*/*CD274* (PD-L1) had significantly higher expression in the high-3′-UTR piSNV ratio groups (Supplementary Fig. 2e, f). Many previous studies have proved that immune checkpoint therapies (such as anti-PD-1/PD-L1 therapies) have extraordinary therapeutic effects in cancer patients and revolutionize the treatment standard for multiple cancers^76^. However, more matched expression data of different cancers are needed to illustrate the immune microenvironment characteristics at the posttranscriptional regulation level. Finally, we identified several potential therapeutic compounds for patients with specific cancer types. Recent studies also show that RBPs play a role in viral infections. The expression of RBP RBM47 is upregulated by Dengue virus (DENV) infection and has an inhibitory effect on DENV replication; RBM47 is an Interferon-stimulated genes (ISG) that is upregulated by multiple viral induction or interferon stimulation and has broad-spectrum antiviral effects. rBM47 had no significant effect on IFN production, but had a significant enhancing effect on the activation of the Interferon (IFN) response element ISRE and the expression of ISGs. In the RBP immunoprecipitation assay, RBM47 specifically bound the 3′-UTR of type I IFN receptor IFNAR1 mRNA, stabilized the mRNA, and subsequently increased the protein level of intracellular IFNAR1, promoted viral infection or interferon-induced phosphorylation of STAT1/2, enhanced interferon-stimulated expression of gene ISGs and amplified the antiviral effect of the host^77^.

Generally, our study revealed the comprehensive characteristics and clinical relevance of 3′-UTR piSNVs across cancers, providing new insight for investigating posttranscriptional regulation, which contributes to tumor progression and may promote the development of new strategies for cancer treatment.

## Methods

### Identification of 3′-UTR piSNVs from somatic mutation WGS data of 18 cancer types

We downloaded somatic mutations derived from WGS data of 2413 patients across 18 cancer types in the PCAWG (https://dcc.icgc.org/releases/PCAWG/) project^17^, including Biliary-AdenoCA, Bone-Osteosarc, Breast-AdenoCa, CNS-Medullo, CNS-PiloAstro, Eso-AdenoCa, Kidney-RCC, Liver-HCC, Lymph-BNHL, Lymph-CLL, Myeloid-MPN, Ovary-AdenoCA, Panc-AdenoCA, Panc-Endocrine, Prost-AdenoCA, Skin-Melanoma, and Stomach-AdenoCA, as well as our in-house ESCC WGS samples (663 patients)^18^. We used WGS samples from the DSMNC (https://dsmnc.big.ac.cn/) database (Database of Somatic Mutations in Normal Cells)^19^ as a control, and we obtained 0.77 million somatic SNVs occurring in over 579 human normal cells from the DSMNC database.

PIVAR algorithm was our previously developed to evaluate the impact of mutations on posttranscriptional regulation^6^. We updated PIVAR algorithm to evaluate the impact of mutations on posttranscriptional regulation by adding the latest 318 eCLIP-seq data from ENCODE (112 RBPs) to identify 3′-UTR piSNVs^38,39^, which could disrupt the binding between RNAs and RBPs. We used the Wilcoxon rank-sum test to compare the distribution of the 3′-UTR piSNV ratio (ratio of 3′-UTR piSNVs to 3′-UTR SNVs) between the control samples and samples of each cancer type. In detail, we calculated the ratio of piSNVs to SNVs in the 3′-UTR for each sample and used Wilcoxon rank-sum test to evaluate the distribution differences of 3′-UTR piSNV ratio between cancer samples in each cancer type and control samples.

GWAS SNPs data was downloaded from GWAS Catalog database^20^ (https://www.ebi.ac.uk/gwas/downloads) and CCGD-ESCC database^21^ (http://db.cbi.pku.edu.cn/ccgd/ESCCdb).

### Clinical survival analysis

Corresponding clinical data of each cancer type was collected from the PCAWG (https://dcc.icgc.org/releases/PCAWG/) project and our in-house ESCC cohort. Then, the ‘surv_cutpoint’ function in ‘survminer’ R package was used to determine the optimal 3′-UTR piSNV ratio based on the OS data of the patients. Overall survival analysis was performed with the Kaplan–Meier method and *p* value was calculated with log-rank test. In the multivariate cox proportional regression analysis, age, sex, tumor-node-metastasis (TNM) stage, and the race of patients was assessed to analyze the relationship between the 3′-UTR piSNV ratio and clinical outcomes.

### Characteristics of immune cell types and immune regulators between the high- and low-3′-UTR piSNV ratio groups

We employed the CIBERSORT^24^ algorithm to calculate the abundance scores of 22 immune cell types to evaluate the cellular heterogeneity landscape in ESCC and Liver-HCC by RNA-seq data. We obtained immune checkpoint genes from previous studies^76,78^ and used the ‘DESeq2’ R package^79^ to calculate the expression difference between the high-3’-UTR piSNV ratio group and the low-3’-UTR piSNV ratio group. We compared the abundance of each immune cell type and gene expression between the high- and low-3’-UTR piSNV ratio groups by the Wilcoxon rank-sum test.

### RBP motif enrichment of 3′-UTR piSNV loci and their impact on RBP binding

Many RBPs interact with mRNAs via a limited set of modular RNA-binding domains, including the RNA recognition motif, heterogeneous nuclear ribonucleoprotein K-homology domain, and zinc fingers^80^. The RNA-binding domains of RBPs initially determine the specificity and preferences of RNA binding with specific sequence motifs^81^. Therefore, we downloaded 247 positional weight matrices (PWMs) of the inferred RNA binding motif from the AURA database^82^, which were used to call motif matches in the transcriptome. RBP motif and CLIP-seq-derived RBP binding were integrated to identify 3′-UTR piSNVs^38,39^; FDR and OR values were calculated with Fisher’s exact tests to identify the significantly enriched RBPs affected by 3′-UTR piSNVs (FDR < 0.05 and OR > 1).

To further estimate the influence of piSNVs on RBP binding, the crystal structures of the PTBP1-RNA (PDB ID: 2AD9) and SRSF1-RNA (PDB ID: 6HPJ) complexes were downloaded from the Protein Data Bank^83^ (https://www.rcsb.org/); the binding strength was predicted with the PRIdictor^35^ webtool (http://bclab.inha.ac.kr/pridictor), and the structures were visualized with PyMOL software (https://pymol.org/).

### Chemiluminescent electrophoresis mobility shift assays

To evaluate the functional effect of RNA mutation on binding of RBP, we used LightShift Chemiluminescent RNA EMSA kit (Catalog # 20158; Thermo Scientific, Rockford, USA) to performe electrophoresis mobility shift assays. RBP PTBP1 kept on −80 °C was used in the assay. Then, 200 ng PTBP1 protein was pre-incubated with 0.2 µL tRNA (10 mg/mL) in 1× RNA EMSA binding buffer containing 5% glycerol for 10 min at room temperature to block unspecific binding as much as possible. After that, 100 fmol synthesized 3′-biotin-labeled wild-type or point-mutated RNA oligos (Supplementary Table 5) for each reaction were respectively added to the mixture to a final volume of 20 µl and incubated for 20 min at room temperature. Then, 1× loading buffer was added to the RBP-RNA mixture and immediately loaded into the pre-run 6% TBE polyacrylamide gel, and ran at 100 V for 45–60 min in cooled 0.5× TBE buffer. Samples were then transferred to positively charged nylon membrane (Catalog # FFN15, Beyotime, Shanghai, China), and crosslinked with UV-light crosslinking instrument equipped with 254 nm bulbs for 5 min. The subsequent blocking, washing and detection were performed according to the manufacturer’s instructions.

### Predicted RNA modification and APA influence on 3′-UTR piSNVs

We annotated RNA modifications and APA changes in 3′-UTR piSNVs with RMVar^51^ and APADB^52^. We collected 22 kinds of APA regulators and 20 kinds of m6A RNA modification regulators, including 7 m6A writers, 2 erasers and 11 readers.

### miRNA annotation of 3′-UTR piSNVs

We identified possible binding miRNAs of 3′-UTR piSNVs with TargetScan v7.2^46^, miRNASNP v3^47^, starBase v3.0^48^, and miRDB v6.0^45^ software, and we built a network of the top miRNAs related to 3′-UTR piSNV-affected genes.

### Differential expression analysis

RNA-seq/miRNA raw read count data of seven types of cancer tissues and corresponding normal tissues were collected from the PCAWG and our in-house ESCC cohorts. The R package ‘DESeq2’^79^ was used to evaluate the expression of 2930 3′-UTR piSNV-affected genes, 248 enriched RBPs, miRNA and m6A/APA regulators identified in the previous steps, and genes with fold-change >2 and FDR < 0.05 were considered to be significant differentially expressed genes.

### Identification of coexisting protein damage-related mutations in 3′-UTR piSNV-affected genes

Functional consequences on proteins of identified 2930 3′-UTR piSNV-affected genes were predicted with activeDriverWGS tool^84^. Then, the 3’-UTR-length of each gene was applied to corrected occurrence frequency of protein damaging mutations and 3’-UTR posttranscriptional impaired mutations in these genes was analyzed in all 2413 cancer patients^85^.

### Functional enrichment and pathological network/pathway analysis

We performed functional enrichment analysis via the ‘clusterProfiler’ R package^65^ with a Bonferroni correction test and identified significant pathways with FDR values < 0.05. Then we used the HotNet2^66^ and activePathway^67^ workflows to mine significantly mutated subnetworks and pathways according to HINT + HI2012 (a combination of the HINT network^68^ and the HI-2012^69^), iRefIndex^70^ and Multinet^71^ protein–protein interaction networks, and the sample frequency of 3′-UTR piSNVs and damage score for the affected genes were taken as the network heat score to identify significant subnetworks and pathways with default parameters.

### Potential clinical drug analysis

To further explore the therapeutic implications of 3′-UTR piSNV-affected genes, drug response signatures assembled in CMAP build 02 (Broad Institute)^74^ were downloaded to compare with the gene expression of identified 3′-UTR piSNV-affected gene, which contains information on gene expression profiles and the sensitivity to 1309 compounds.

### Reporting summary

Further information on research design is available in the Nature Research Reporting Summary linked to this article.

## Supplementary information

supplementary figures

supplementary tables

Reporting Summary

## Data Availability

The majority of the datasets analyzed in this study were downloaded from the Pan-Cancer Analysis of Whole Genomes (PCAWG) study (https://dcc.icgc.org/releases/PCAWG/); NGS and clinical data of ESCC samples are available in Genome Sequence Archive (GSA) (https://ngdc.cncb.ac.cn/gsa-human/) under accession HRA000021; Somatic mutations in normal cells were downloaded from the DSMNC database (https://dsmnc.big.ac.cn/).

## Code availability

PIVar algorithm are available at GitHub (https://github.com/WeiWenqing/PIVar). All computational tools used with default parameters in this study are available for download as open-source software and are detailed in “Methods”.
