# Supplementary material for: Comprehensive characterization of posttranscriptional impairment-related 3′-UTR mutations in 2413 whole genomes of cancer patients
Source: NPJ Genom Med. 2022 Jun 2;7:34. doi: 10.1038/s41525-022-00305-0 (PMC9163142; doi:10.1038/s41525-022-00305-0)
Supplement: Supplementary file 1 — supplementary figures [file 41525_2022_305_MOESM1_ESM.pdf]

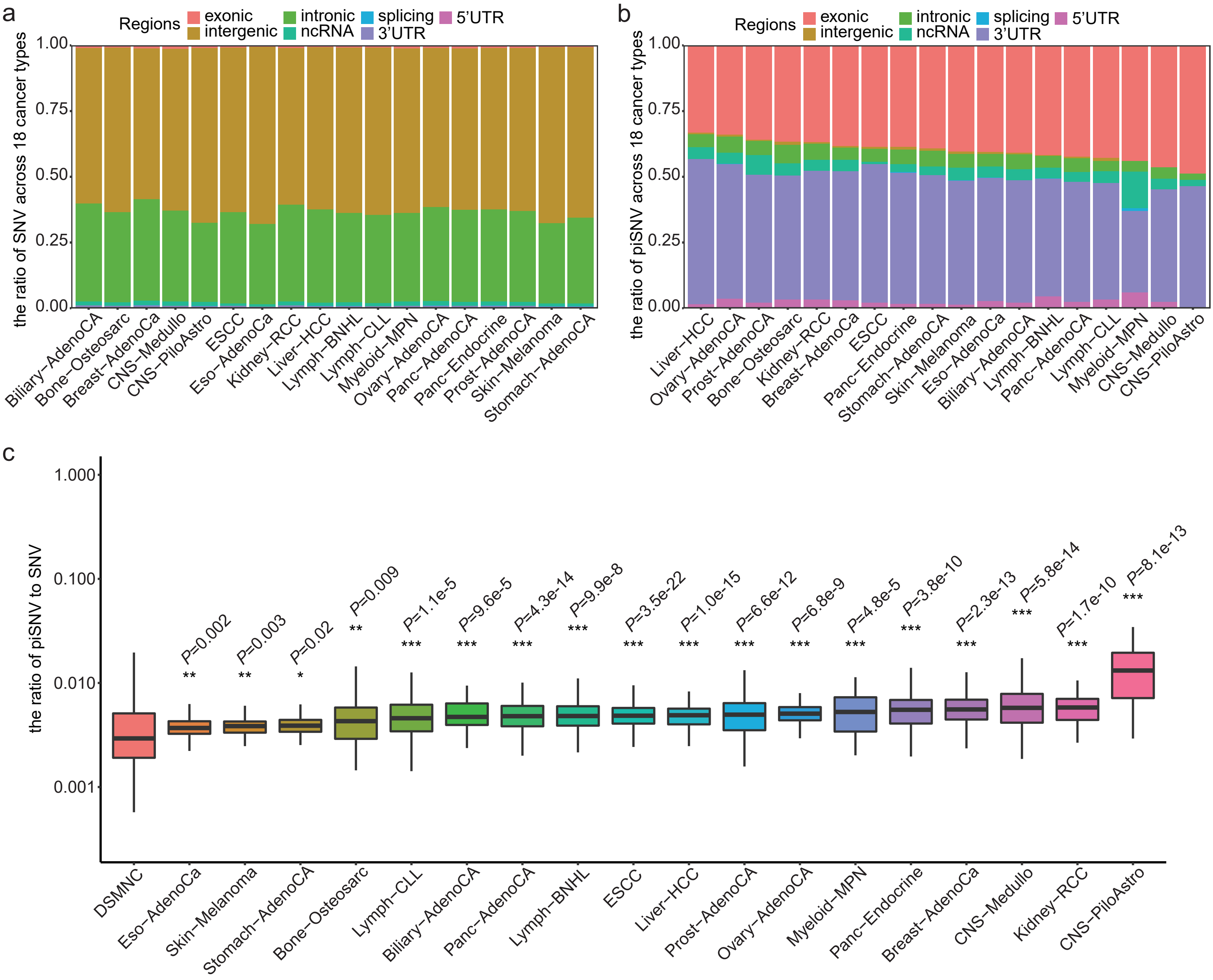

Supplementary Figure 1 Somatic piSNVs identified in PCAWG project and ESCC cohorts. Somatic mutational region of SNVs (**a**) and post-transcriptional impaired SNVs (piSNVs) (**b**) identified in PCAWG project and ESCC cohorts. **c** Compared to controls in the DSMNC database, the proportion of somatic whole-genome piSNVs was significantly elevated in the PCAWG project and ESCC cohorts (* P<0.05, ** P<0.01, *** P<0.001). We calculated the ratio of piSNV to SNV for each sample, and used wilcoxon rank-sum test to evaluate the distribution differences of piSNV ratio between cancer samples in each cancer type and control samples. Boxplots elements represent: center line = median, upper and lower hinges = 25 and 75% percentiles, upper and lower whisker = mean ± 1.5*IQR.

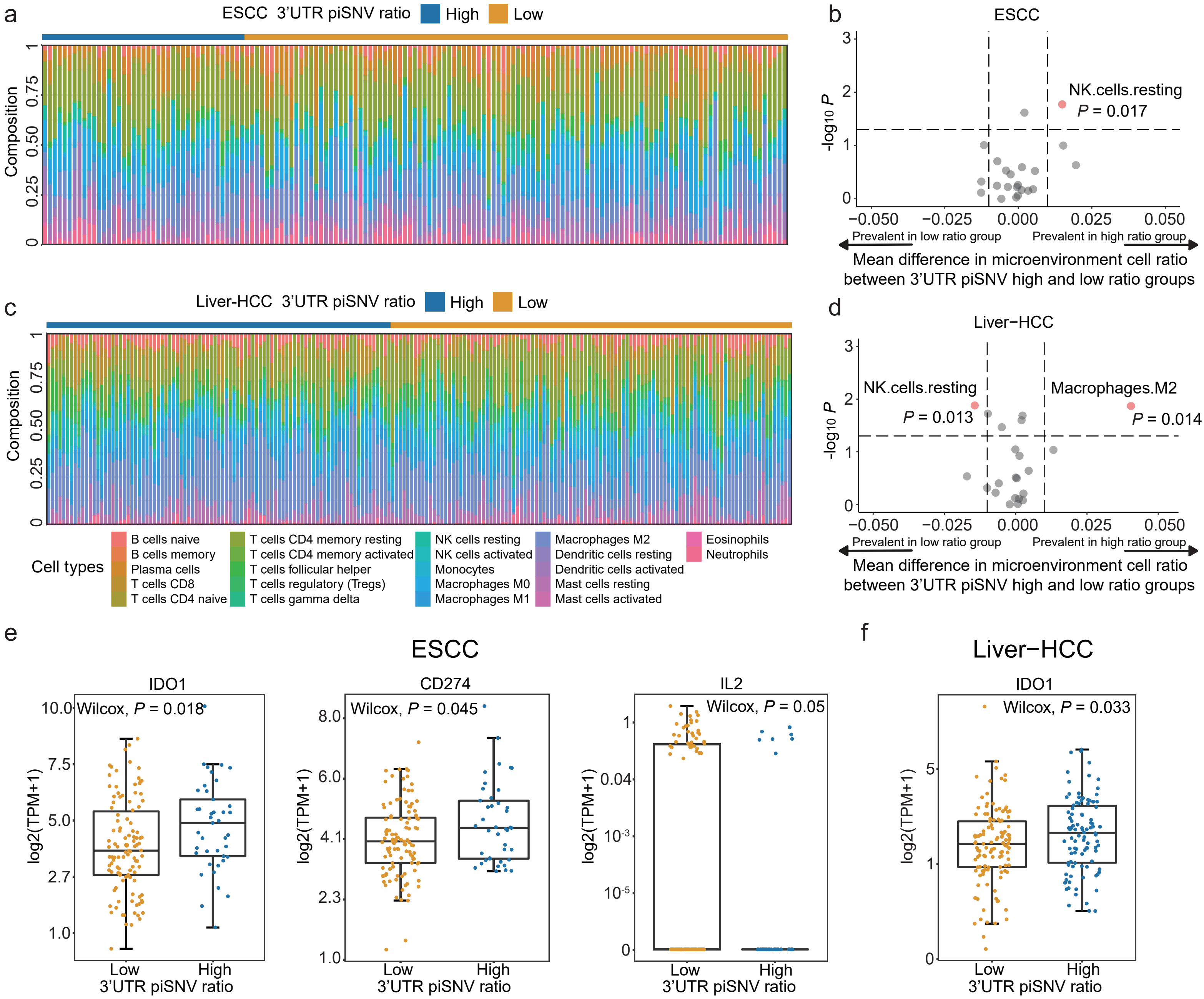

Supplementary Figure 2 Immune microenvironment characteristics related to the 3'-UTR piSNV ratio. The relative abundance of the 22 types of immune cells in ESCC (**a, b**) and Liver-HCC (**c, d**), red points represent the immune cells that had p value below 0.05 and mean difference in microenvironment cell ratio between 3'-UTR piSNV high and low ratio groups over 0.01 (Wilcoxon rank-sum test). Tumor expression of IDO1, CD274 and IL2 in ESCC (**e**) and IDO1 in Liver-HCC (**f**). Boxplots elements represent: center line = median, upper and lower hinges = 25 and 75% percentiles, upper and lower whisker = mean ± 1.5*IQR.

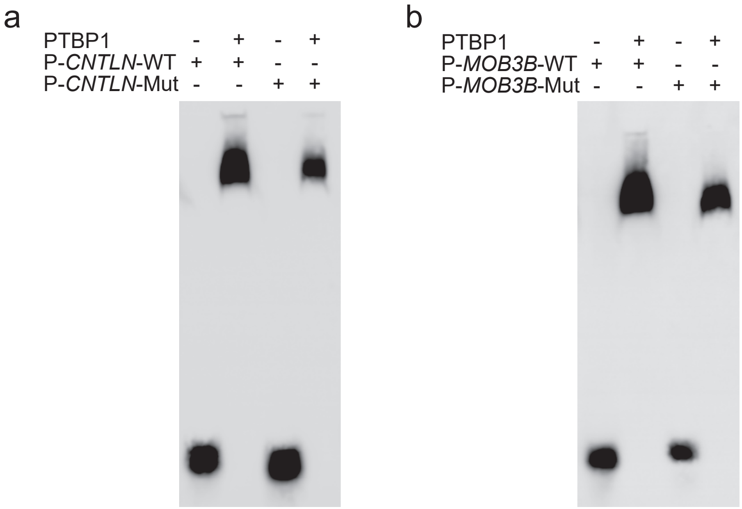

c

PRIdictor_score	1-C	2-U	3-C	4-U	5-C	6-U
2AD9(5'-CUCUCU-3')	0.59	0.80	0.75	0.87	0.85	0.66
2AD9_4'U/A	0.87	0.92	0.87	0.83	0.61	0.41
2AD9_5'C/T	0.71	0.90	0.79	0.84	0.80	0.38
2AD9_5'C/A	0.62	0.91	0.83	0.84	0.85	0.46

d

PRIdictor_score	1-A	2-A	3-C	4-A	5-A	6-A
6HPJ(5'-AACAAA-3')	0.79	0.79	0.77	0.69	0.54	0.5
6HPJ_6'A/U	0.71	0.64	0.69	0.62	0.49	0.47

Supplementary Figure 3 The binding impacts of 3'-UTR piSNVs on RBP binding.
Electrophoretic mobility shift assays (EMSA) results show the binding impact of 3'-UTR piSNVs of CNTLN (**a**) and MOB3B (**b**) on the recognition of PTBP1 to their RNA targets. Binding scores of the PTBP1 (**c**; PDB ID: 2AD9) or SRSF1 (**d**; PDB ID: 6HPJ)-RNA complex were performed by PRIdictor webtool, 0.5 was regarded as classification threshold between binding and non-binding.

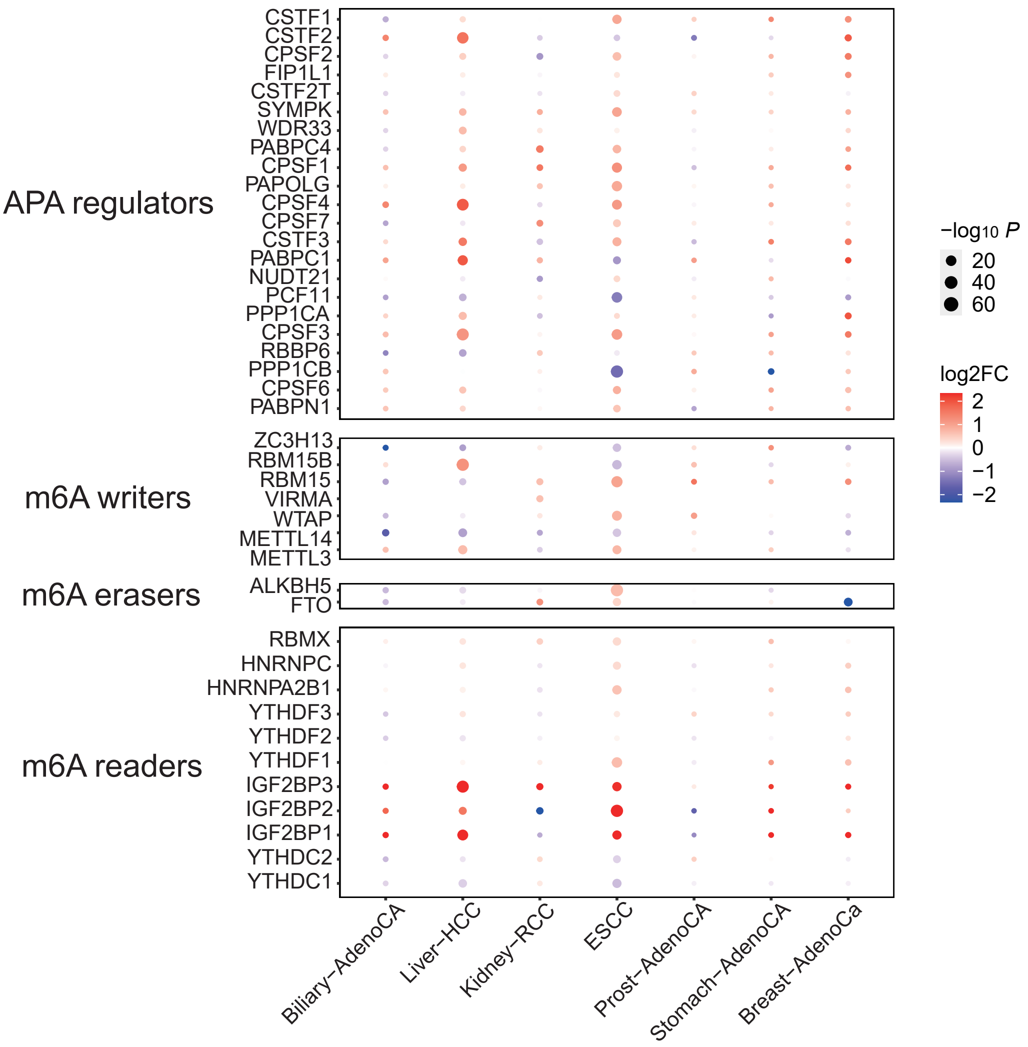

Supplementary Figure 4 Expression of APA regulators and m6A regulators (m6A writers/m6A erasers/m6A readers) in different cancer types.
